# Protocol for evaluating the abilities of diverse nitroaromatic prodrug metabolites to exit a model Gram negative bacterial vector

**DOI:** 10.1016/j.mex.2020.100797

**Published:** 2020-01-23

**Authors:** Jasmine V.E. Chan-Hyams, David F. Ackerley

**Affiliations:** a School of Biological Sciences, Victoria University of Wellington, Wellington, 6012, New Zealand; b Centre for Biodiscovery, Victoria University of Wellington, Wellington, 6012, New Zealand

**Keywords:** BDEPT, GDEPT, Cancer gene therapy, Nitroreductase, Bystander effect, CB1954, Metronidazole

## Abstract

Bacterial-directed enzyme-prodrug therapy (BDEPT) uses tumour-tropic bacteria armed with a genetically-encoded prodrug-converting enzyme to sensitise tumours to a systemically-administered prodrug. A strong bystander effect (i.e., efficient bacteria-to-tumour transfer of activated prodrug metabolites) is critical to maximise tumour cell killing and avoid bacterial self-sterilisation. To investigate the bystander effect in bacteria we developed a sensitive screen that utilised two *Escherichia coli* strains grown in co-culture. The first of these was an activator strain that overexpressed the *E. coli* nitroreductase NfsA, and the second was a nitroreductase null recipient strain bearing an SOS-GFP DNA damage responsive gene construct. In this system, induction of GFP by genotoxic prodrug metabolites can only occur following their transfer from the activator to the recipient cells. This can be monitored both in fluorescence based microtitre plate assays and by flow-cytometry, enabling modelling of the abilities of diverse nitroaromatic prodrug metabolites to exit a Gram negative vector.

## Graphical abstract

**Figure:**
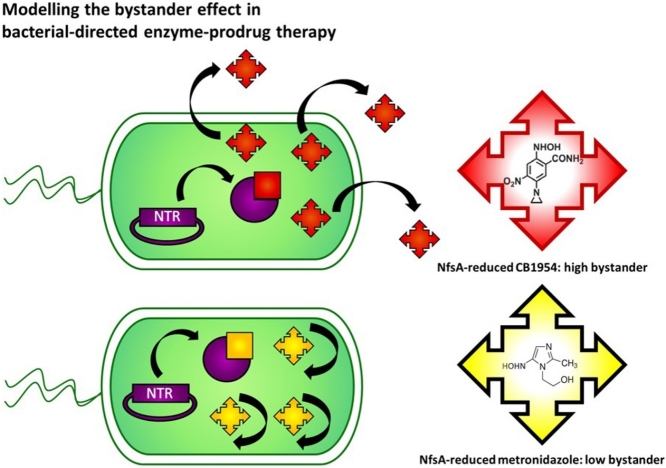

**Specification Table**

**Table:** 

Subject Area:	Pharmacology, Toxicology and Pharmaceutical Science
More specific subject area:	Cancer gene therapy
Protocol name:	Protocol for evaluating the abilities of diverse nitroaromatic prodrug metabolites to exit a model Gram negative bacterial vector
Reagents/tools:	***Reagents*** (supplier unimportant) • High-grade sterile LB • Antibiotics for maintenance of plasmids (ampicillin and spectinomycin in the examples herein) • IPTG • Sterile culture vials/tubes for cell growth • Sterile 384-well microtitre plates • Shaking incubator for culture growth ***Equipment*** (supplier unimportant) • Spectrophotometer for monitoring cell growth • Microplate photometer for monitoring cell growth and fluorescence • Flow cytometer for monitoring fluorescence of individual bacteria • Software appropriate for analysis of flow cytometry data ***Bacterial strains, genes and plasmids*** (dependent on model system used) • *Activator strain* - expresses the prodrug activating enzyme but does not report on presence of the active prodrug metabolite. • *Recipient strain -* does not express the prodrug activating enzyme but can report on receipt of the active prodrug metabolite. • *Negative control strain* - does not express the prodrug activating enzyme and does not report on presence of the active prodrug metabolite (used in co-culture with the recipient strain to control for baseline levels of signal generated by the prodrug acting on the recipient strain). • *Positive control strain -* expresses the prodrug converting enzyme and can report on receipt of the active prodrug metabolite (used to set fluorescence gates during flow cytometry and to optimise the prodrug concentration(s) used in the assay).
Experimental design:	Protocol for evaluating the ability of activated prodrug metabolites to exit a bacterial activator cell and cause damage to surrounding cells, by co-culture of an activating strain with a recipient strain that lacks the activating enzyme, but carries a reporter construct able to quantify the damage.
Trial registration:	N/A
Ethics:	N/A

**Value of the Protocol**

**Table:** 

• Direct relevance to BDEPT - First assay to measure the ability of prodrug metabolites to exit a model bacterial vector rather than tumour activator cells. • Affordability and ease of use. • Miniaturised - Assay optimised for 384-well microtitre plates, reducing synthesis costs for analysis of bespoke prodrug candidates.

## Description of protocol

The bystander effect (i.e., the ability of activated prodrug metabolites to transfer from activating cells to neighbouring cells) is essential to bacterial-directed enzyme-prodrug therapy (BDEPT), as failure of the prodrug to exit the activating bacterium will negate the therapy. We present here the first protocol to quantify the ability of activated prodrugs to exit a bacterial activator cell, based on co-culture of an activator strain expressing a prodrug converting enzyme with a recipient strain that can quantify drug-induced damage. Both flow cytometry and microplate methodologies are described.

### Background

The ability of activated prodrug metabolites to transfer from activating cells to neighbouring cells (*i.e.,* the bystander effect) is a critical aspect of enzyme-prodrug therapies [1]. For viral-directed enzyme-prodrug therapy (VDEPT), transfer of prodrug metabolites from activating cells to untransfected adjacent cells has been modelled using mixed multilayer human cell cultures [2]. The bystander effect is even more important in bacterial-directed enzyme-prodrug therapy (BDEPT) where failure of the prodrug to exit the activating bacterium will negate the therapy entirely. However, an effective model to assay the abilities of different prodrugs to exit an activating bacterial cell has not previously been described. To address this, we developed a model that employs bacterial activator cells (over-expressing a prodrug-converting nitroreductase) in co-culture with nitroreductase-null SOS-GFP recipient cells (able to report on the levels of DNA damage induced via the bystander effect of activated nitroaromatic genotoxins [3]. As the model required considerable optimisation, we describe here a detailed protocol to enable implementation of the model in any laboratory with fluorescent plate-reader or flow cytometry capabilities.

### Materials

• High-grade sterile LB
• Antibiotics (for maintenance of plasmids; for us these were ampicillin and spectinomycin)
• IPTG
• Sterile culture vials/tubes for cell growth
• Sterile 384-well microtitre plates
• Shaking incubator for culture growth
• Spectrophotometer for monitoring cell growth
• Microplate photometer for monitoring cell growth and fluorescence
• Flow cytometer for monitoring fluorescence of individual bacteria
• Software appropriate for analysis of flow cytometry data

### Bacterial strains, genes and plasmids

• *Activator strain* - expresses the prodrug activating enzyme but does not report on presence of the active prodrug metabolite.
• *Recipient strain -* does not express the prodrug activating enzyme but can report on receipt of the active prodrug metabolite.
• *Negative control strain* - does not express the prodrug activating enzyme and does not report on presence of the active prodrug metabolite (used in co-culture with the recipient strain to control for baseline levels of signal generated by the prodrug acting on the recipient strain).
• *Positive control strain -* expresses the prodrug converting enzyme and can report on receipt of the active prodrug metabolite (used to set fluorescence gates during flow cytometry and to optimise the prodrug concentration(s) used in the assay).

In our previous study [3] and henceforth throughout this protocol, the prodrug converting enzymes were bacterial nitroreductases. The nitroreductase genes were PCR-amplified from genomic DNA stocks and cloned into the ampicillin resistant expression plasmid pUCX as previously described [4,5]. Our primary focus was the *E. coli* nitroreductase NfsA, which exhibits high level activity with a broad range of nitroaromatic substrates [6].

Our *activator strain* and *recipient strain* were the *E. coli* strains 7NT and SOS-R4 as previously described [7]. These are isogenic derivatives of the *E. coli* strain W3110 with seven endogenous nitroreductase candidate genes (*nfsA, nfsB, azoR, nemA, mdaB, yieF and ycaK*) deleted to reduce background activation of nitroaromatic prodrugs together with deletion of the *tolC* transporter gene to restrict active efflux of unreduced prodrug. The 7NT activator strain was transformed with pUCX expressing a prodrug-converting nitroreductase, and additionally transformed with empty pCDFDuet plasmid to confer spectinomycin resistance. The recipient strain was SOS-R4 (7NT pre-transformed with pANODuet, a spectinomycin resistant plasmid expressing a *gfp* reporter gene under control of the SOS-responsive *sfiA* promoter), further transformed with empty pUCX to confer ampicillin resistance. The *negative control strain* was 7NT transformed with empty pUCX; and the *positive control strain* was SOS-R4 transformed with pUCX expressing the prodrug-converting nitroreductase.

### Optimizing prodrug concentration

To accurately model the bystander effect in bacteria, the prodrug concentration must be optimised to ensure acceptable growth inhibition of the positive control strain. We have previously found that a greater than 20 % decrease in culture turbidity in response to prodrug challenge, relative to an unchallenged control, can cause a reduction in output during SOS assays [4]. To enable comparison of multiple prodrugs, use empirical testing to determine concentrations of each prodrug that elicit comparable levels of reporter gene expression from the positive control strain, without causing more than 20 % growth inhibition. In our assays, the prodrug challenge time was typically 3.5−4 h to generate a measurable difference in GFP fluorescence signal between the prodrug challenged and unchallenged conditions in a 384-well microtitre assay using the SOS-R4 strain. However for non-nitroaromatic prodrugs this incubation time may need to be optimised. For nitroreductase assays metronidazole has a negligible bacterial bystander effect [3] and can be used as a nil bystander control to compare to other prodrugs.

## Method

### Bacterial bystander assays performed in 384 well microtitre plates

**Fig. 1:**
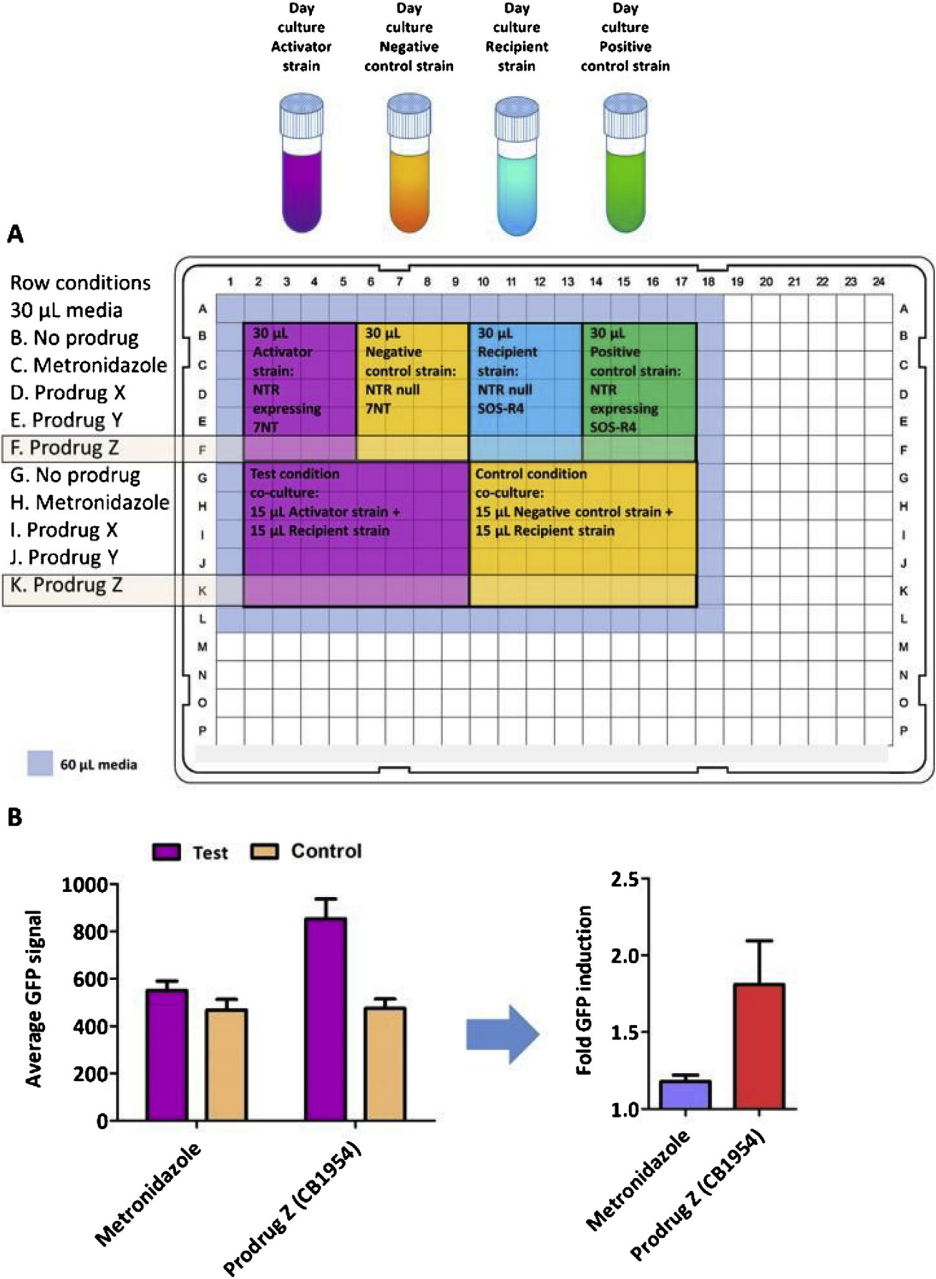
**A. Suggested schematic for the bacterial bystander assay performed in a microplate.** The SOS response, induced by transfer of activated prodrug metabolites from nitroreductase-expressing (NTR) activator cells to nitroreductase null SOS-R4 recipient cells, was measured by mean GFP fluorescence recorded over an entire population of *E. coli*. In this example a 50:50 mixed co-culture of nitroreductase-expressing 7NT activator cells and SOS-R4 recipients was incubated with either no prodrug, 5 μM metronidazole, or empirically optimised amounts of hypothetical prodrug X, hypothetical prodrug Y or prodrug Z (in this example, 50 μM CB1954) at 200 rpm, 30 °C for 3.5 h, after which GFP fluorescence (excitation 490 nm/emission 530 nm) was measured. The total volume of all cultures in the microtitre assay was 60 μL. A protective barrier of 60 μL of sterile media was dispensed into the immediately surrounding wells. **B. Measuring the bacterial bystander effect by calculating the fold increase in fluorescence resulting from cell-to-cell transfer of activated prodrug metabolites.** To calculate the fold difference in fluorescence, average the raw fluorescence units of each technical replicate performed for the Test condition (purple bar) and then divide by the average fluorescence of the technical replicates performed for the corresponding Control condition (tan bar). Error bars represent the standard deviation of the average fold increase in GFP induction across a minimum of three biological replicates, each derived from an independent overnight culture. A fold increase in fluorescence >>1 indicates a high bacterial bystander effect was detected, as per Prodrug Z (CB1954) (red bar) while a fold increase in GFP close to 1 indicates a poor bacterial bystander effect as per metronidazole (blue bar).

Evaluation of cell-to-cell transfer of activated prodrug metabolites in *E. coli* can be performed in 384 well microtitre plates (Fig. 1).

• *Test condition:* co-culture of activator strain (nitroreductase-expressing 7NT) and recipient strain (nitroreductase null SOS-R4) challenged with the prodrug.
• *Control condition:* co-culture of negative control strain (nitroreductase null 7NT) and recipient strain (nitroreductase null SOS-R4) that are together challenged with the prodrug.

## Procedure

1 Overnight cultures of a nitroreductase-expressing 7NT activator strain, a nitroreductase null 7NT control strain, and the SOS-R4 recipient strain were inoculated in 3 mL aliquots of LBASG media (lysogeny broth supplemented with 100 μg ml^−1^ ampicillin, 50 μg ml^−1^ spectinomycin and 0.2 % glucose (w/v)) were established in sterile 15 ml centrifuge tubes and incubated overnight for 16 h at 30 °C with shaking at 200 rpm.
2 The following morning, cultures were visually assessed to ensure each had achieved expected levels of turbidity (spectrophotometric measurement not essential at this stage, but if performed the OD_600_ should exceed 2.5). Next, 0.5 mL of each overnight culture were used to inoculate distinct day cultures of 10 mL of LBASGI (LBASG supplemented with 50 μM IPTG, to induce nitroreductase expression), which were then incubated at 30 °C, 200 rpm for 3 h. Prior to prodrug challenge all cultures were diluted using fresh LBASGI media to an OD_600_ of 0.8.
3 In 384-well microtitre plates, 4–8 wells were filled with either 30 μL control media (LBASGI only) or 30 μL challenge media (LBASGI supplemented with twice the desired final challenge concentration of the prodrug). See Fig. 1A for a suggested 384-well microtitre plate design.
  a For example, to establish an activator to recipient ratio of 50:50
    i Test condition: pipette 15 μL of the nitroreductase-expressing 7NT activator day culture into the assigned wells.
    ii Control condition: pipette 15 μL of the nitroreductase null 7NT negative control day culture into the assigned wells.
    iii Pipette 15 μL of the nitroreductase null SOS-R4 recipient strain day culture into all of these wells to bring the final volume to 60 μL per well.
  b For example, to establish an activator to recipient ratio of 10:90
    i Test condition: pipette 100 μL of the nitroreductase-expressing 7NT activator day culture into a 1.5 mL centrifuge tube.
    ii Control condition: pipette 100 μL of the nitroreductase null 7NT negative control day culture into a 1.5 mL centrifuge tube.
    iii Add 900 μL of nitroreductase null SOS-R4 recipient strain day culture to each tube and mix well by pipetting.
    iv Dispense 30 μL into each of the assigned wells to bring the final volume to 60 μL per well.
  c For each strain, grow replicate cultures in both control and challenge media to enable background levels of growth inhibition and GFP expression (recipient strain only) to be ascertained.
    i For each of the three strains, pipette 30 μL of day culture into separate wells containing 30 μL control or 30 μL challenge media.
  d A protective barrier of 60 μL of sterile media was dispensed into the immediately surrounding wells to prevent uneven exposure to incubation conditions, which can influence the growth of cultures.
  e After dispensing all the day cultures into the microtitre plate, mix well by pipetting.
  f Record the OD_600_ for all experimental wells on a microplate photometer.
  g Incubate the microtitre plate at 30 °C, 200 rpm for 3.5 h, after which the OD_600_ and GFP fluorescence (excitation 490 nm/emission 530 nm) should be recorded on a microplate photometer.
4 To calculate the fold increase in fluorescence resulting from cell-to-cell transfer of activated prodrug metabolites (Fig. 1B).
  a Derive the mean fluorescence of the technical replicates for the Test condition and divide this by the mean fluorescence of the technical replicates for the corresponding Control condition.
  b Assess growth inhibition (OD_600_ for prodrug challenged *versus* unchallenged conditions) to ensure that the level of inhibition does not exceed 20 %.
5 In this microtitre assay, biological replicates are derived from independent overnight cultures. Technical replicates are replicate microtitre cultures derived from a single overnight culture for each condition.

### Bacterial bystander assays performed using flow cytometry

**Fig. 2:**
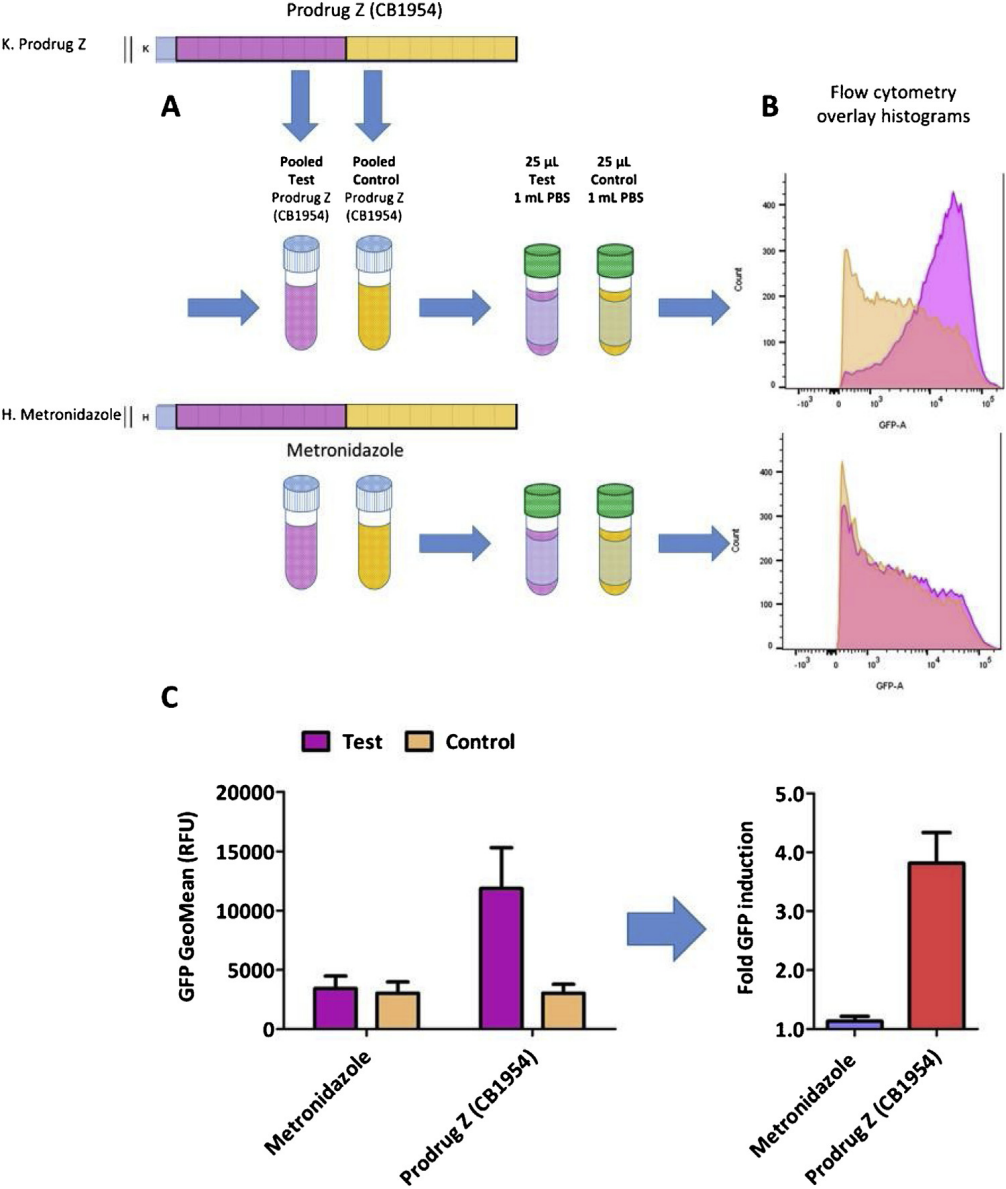
**A. Schematic of the bacterial bystander assay as performed by flow cytometry.** The SOS response, induced by transfer of activated prodrug metabolites from nitroreductase-expressing 7NT activator cells to nitroreductase null SOS-R4 recipient cells, was measured by GFP fluorescence recorded in individual bacterial cells by flow cytometry. Eight microtitre replicates, as per Fig. 1, were pooled and 25 μL of each pooled sample was measured for GFP fluorescence using flow cytometry. In this example 50:50 mixed co-culture of nitroreductase-expressing (NTR) 7NT activator cells and SOS-R4 recipients were incubated with either no prodrug, 5 μM metronidazole, or 50 μM prodrug Z (CB1954 in this example). **B. Overlay histograms of test and control conditions for prodrug Z and metronidazole.** Overlay histograms represent technical flow cytometry replicates that indicate the population fluorescence in the selection gate for the mixture containing the nitroreductase-expressing 7NT activator cells (Test condition; pink) and the nitroreductase null 7NT strain (Control condition; orange). A large population shift between the GFP signal recorded in the Test and Control indicates a large bystander effect, as observed with Prodrug Z (CB1954), whereas a minimal population shift indicates a poor bystander effect, as observed with metronidazole. **C. Measuring the bacterial bystander effect by calculating the fold increase in fluorescence resulting from cell-to-cell transfer of activated prodrug metabolites.** The average GFP geometric mean (GFP Geomean) for at least three technical replicates represents one biological replicate data point. Biological replicates are derived from independent overnight cultures. Error bars represent the standard deviation of the average fold increase in GFP induction across a minimum of three biological replicates. A fold increase in GFP induction significantly greater than 1 for the Test relative to the Control indicates a high bacterial bystander effect, as with Prodrug Z (red bar), while a fold increase in GFP close to 1 indicates a poor bacterial bystander effect as with metronidazole (blue bar).

Evaluation of cell-to-cell transfer of activated prodrug metabolites in a population of *E. coli* can be performed using flow cytometry immediately following the microtitre plate assay (Fig. 2). Note that two additional sets of replicates must be established in the microtitre plate: one comprising 100 % positive control cells, challenged with prodrug as per the Test condition; and one comprising 100 % negative control cells without prodrug challenge. These replicates will enable the upper and lower limits of the selection gates to be set.

Conditions to set fluorescence selection gates:

• *Negative control strain* (nitroreductase null 7NT) in LBASGI media without prodrug. Use to set lower limit of selection gate to remove background auto fluorescence.
• *Positive control strain* (nitroreductase-expressing SOS-R4) expresses the prodrug converting enzyme. Use to set upper limit of selection gate to capture the highest expected fluorescence in co-cultures.

## Procedure

1 Pool each set of technical replicates for each challenge condition from the microtitre plate into individual micro centrifuge tubes (Fig. 2A).
  a Make sure to include the individually challenged negative and positive control strains to establish the lower and upper flow cytometer parameters respectively.
2 Prepare 14 mL round-bottom polypropylene tubes (or tubes appropriate to the flow cytometer you have access to) by dispensing 1 mL of filter sterilized Phosphate Buffered Saline pH 7.4 (PBS buffer) per tube.
3 Setting up flow cytometer
  a Set to lowest flow rate available to more accurately assess fluorescence of the individual bacteria.
  b Pipette 25 μL of the pooled no-prodrug cultures for the negative control strain into one of the polypropylene tubes containing PBS and mix by vortexing for five seconds.
    i Begin sample collection on flow cytometer
    ii Adjust the FSC and SSC values until you can identify the bulk of the bacterial population in the real-time dot plot display.
  c Set selection gate using histograms
    i Pipette 25 μL of the pooled no-prodrug cultures for the negative control strain into one of the polypropylene tubes containing PBS and mix by vortexing for five seconds.
    ii Begin sample collection and establish the level of background fluorescence owing to *E. coli* auto-fluorescence. Set the selection gate immediately above this boundary.
    iii Select the prodrug conditions you are going to collect data for. Pipette 25 μL of the pooled culture sample for the positive control strain challenged with that prodrug into one of the polypropylene tubes containing PBS and mix by vortexing for five seconds.
    iv The positive control strain is used to set the upper limit of the selection gate to capture the highest expected fluorescence in co-cultures.
    v Adjust the GFP parameters of the flow cytometer until the cells with the highest GFP fluorescence in the population are captured and displayed in the dot plot and histogram. An upper limit is achieved when no significant number of cells is captured beyond this GFP parameter.
    vi For each new prodrug tested, reset the upper limit of the selection gate to the upper-most GFP signal recorded for the positive control strain challenged with that prodrug.
  d On a Becton Dickinson FACSCanto II (BD Biosciences San Jose, CA) our typical parameters were FSC 570, SSC 450 and GFP 555. All data was collected within 2.5 h of removal from the incubator for each biological replicate, using the lowest flow rate of approximately 12 μL of sample per minute (BD LSR II User’s Guide).
4 Collecting data for the Test and Control conditions
  a For the pooled technical replicates of each prodrug challenge condition, pipette 25 μL of the pooled culture sample into one of the polypropylene tubes containing PBS and mix by vortexing for five seconds. For each sample collect 30,000 fluorescent events within the selection gate using a low flow rate while recording all data. It is advisable to also monitor the proportion of events recorded outside the selection gates; if this proportion is substantial then it may be necessary to re-set the selection gate boundaries.
  b Monitor the geometric mean of the GFP fluorescence for all events within the selection gate.
  c Perform this sample collection on the flow cytometer on two more 25 μL aliquots of the pooled sample, to generate three technical flow cytometry replicates for each Test and Control condition.
    i Monitor and record overlap in the selection gate for the technical replicates using overlay histograms (Fig. 2B).
5 Processing the flow cytometry data
  a First review the exported data from the flow cytometer and record the individual GFP geometric mean for each technical flow cytometry replicate using appropriate software. We suggest one of the following programs:
    i Flowing Software version 2.5.1 by Perttu Terho (Turku Centre for Biotechnology, Turku Finland).
    ii Flowjo (© FlowJo, LLC, 2013–2018, BD Biosciences San Jose, CA).
  b To generate each biological replicate, calculate the fold difference in GFP geometric mean of the Test condition versus the Control condition, for the events recorded within the selection gates (Fig. 2C).
    i Average the geometric mean recorded from the three technical replicates of the Test condition then divide this by the average geometric mean recorded from the three technical replicates of the Control condition. Repeat for each prodrug tested.
    ii Values significantly >1 indicate activation of fluorescence in the recipient cell population as a consequence of the bystander effect.
  c We typically performed at least three biological replicates, derived from independent overnight cultures, for each prodrug condition under investigation.

## Additional information

In previous work we have described our development of a GFP-SOS reporter strain to quantify activation of nitroaromatic prodrugs by an over-expressed bacterial nitroreductase [7] and our application of this system to enable directed evolution of superior nitroreductase variants [8]. Here we detail how this system can be adapted to detect activation of prodrugs by non-reporter strains in co-culture, followed by transfer of activated prodrug metabolites to a recipient reporter strain. This bacterial bystander assay is robust, economical and easily implemented. As it can be performed in 384 well microtitre plates, prodrug consumption is low. Importantly, our model informs directly on the relative abilities of prodrug metabolites to exit Gram negative bacterial activator cells. Using this technique we have observed that prodrugs can differ in their suitability for VDEPT versus BDEPT applications [3], which highlights the importance of evaluating enzyme-prodrug combinations in models relevant to the intended GDEPT vector. Taking into account the fundamental physiological differences associated with different gene-delivery systems will facilitate the identification of optimal enzyme-prodrug combinations for each setting as new vectors and prodrugs are developed.

## Declaration of Competing Interest

The authors declare that they have no known competing financial interests or personal relationships that could have appeared to influence the work reported in this paper.
